# A tomographic microscopy-compatible Langendorff system for the dynamic structural characterization of the cardiac cycle

**DOI:** 10.3389/fcvm.2022.1023483

**Published:** 2022-12-22

**Authors:** Hector Dejea, Christian M. Schlepütz, Natalia Méndez-Carmona, Maria Arnold, Patricia Garcia-Canadilla, Sarah L. Longnus, Marco Stampanoni, Bart Bijnens, Anne Bonnin

**Affiliations:** ^1^Paul Scherrer Institute, Villigen, Switzerland; ^2^Institute for Biomedical Engineering, University and ETH Zürich, Zurich, Switzerland; ^3^Department of Cardiac Surgery, Inselspital, Bern University Hospital, Bern, Switzerland; ^4^Department for BioMedical Research, University of Bern, Bern, Switzerland; ^5^BCNatal-Barcelona Center for Maternal-Fetal and Neonatal Medicine, Hospital Sant Joan de Déu and Hospital Clínic, University of Barcelona, Barcelona, Spain; ^6^Cardiovascular Diseases and Child Development, Institut de Recerca Sant Joan de Déu, Esplugues de Llobregat, Spain; ^7^Institut d'Investigacions Biomèdiques August Pi i Sunyer (IDIBAPS), Barcelona, Spain; ^8^Institution for Research and Advanced Studies (ICREA), Barcelona, Spain

**Keywords:** synchrotron, tomographic microscopy, Langendorff, 4D imaging, cardiac cycle

## Abstract

**Introduction:**

Cardiac architecture has been extensively investigated *ex vivo* using a broad spectrum of imaging techniques. Nevertheless, the heart is a dynamic system and the structural mechanisms governing the cardiac cycle can only be unveiled when investigating it as such.

**Methods:**

This work presents the customization of an isolated, perfused heart system compatible with synchrotron-based X-ray phase contrast imaging (X-PCI).

**Results:**

Thanks to the capabilities of the developed setup, it was possible to visualize a beating isolated, perfused rat heart for the very first time in 4D at an unprecedented 2.75 μm pixel size (10.6 μm spatial resolution), and 1 ms temporal resolution.

**Discussion:**

The customized setup allows high-spatial resolution studies of heart architecture along the cardiac cycle and has thus the potential to serve as a tool for the characterization of the structural dynamics of the heart, including the effects of drugs and other substances able to modify the cardiac cycle.

## 1. Introduction

The cardiomyocytes are the contractile cellular units of the myocardium. Within a surrounding fibrous matrix, they aggregate into a complex three-dimensional (3D) mesh with predominant orientations. However, there is still controversy on the exact mode in which individual cardiomyocytes are arranged in 3D. The cardiomyocytes' aggregation and orientation within the ventricular walls determines the propagation of electrical excitation and the force generated by the heart. Therefore, a detailed description is required to achieve a complete understanding of the cardiac cycle and the alterations caused by cardiac remodeling (1, 2). In this context, high resolution four-dimensional (4D) imaging techniques are paramount to assess the structural dynamics of the heart and investigate how cardiomyocyte orientation evolves along the cardiac cycle.

Cardiac architecture has been investigated *ex vivo* using a wide range of imaging techniques, including ultrasound, several optical microscopy techniques, magnetic resonance (MRI) and X-ray imaging (i.e., micro-CT and synchrotron-based X-PCI) (3). However, to study the heart as a dynamic system, *in vitro* (based on isolated heart perfusion) and *in vivo* studies are necessary.

*In vitro* preparations are based on so-called isolated, perfused heart systems. These consist of a series of pumps, tubing and glassware that keep hearts beating outside the donor thanks to the use of specific perfusate, temperature, and oxygenation conditions. Within these systems, Langendorff preparations are based on aortic retrograde perfusion, in which the aortic valve is kept shut, directing the perfusate into the coronary vasculature (4–6).

Among the mentioned imaging techniques, ultrasound and MRI have already been used to investigate how the cardiomyocyte orientation changes along the cardiac cycle. Ultrasound-based techniques, such as shear wave and 3D back-scatter tensor imaging, have been applied in *in vitro* and *in vivo* adult mammals (7, 8). These techniques are cheap and have very high temporal resolution, but lack the spatial resolution to assess cardiomyocyte arrangement at cellular scale. MRI, and more specifically diffusion tensor imaging (DTI) and $T_{2}^{*}$ imaging, has been also applied *in vitro* in isolated, perfused rat hearts (9–15), in rabbit hearts (16, 17) and *in vivo* in pig and human studies (18–31). While allowing whole heart field of views, both spatial and temporal resolution are insufficient to describe the cardiac cycle in detail at the cardiomyocyte-level. Additionally, these techniques often require heart fixation and/or the use of gadolinium-based contrast agents.

On the other hand, synchrotron-based X-PCI has been widely used in the last years for the *ex vivo* study of heart architecture. Grating interferometry (GI) and propagation-based (PB) have been the main X-PCI techniques used. GI has been applied to mice (32), fetal and neonatal human hearts (33–35), while PB X-PCI has been widely used in healthy and pathological cases of rodents (36–39), fetal human hearts (40) and pieces or whole adult human hearts (41–47). While GI allows to obtain three different types of images at once (absorption, differential phase contrast and dark-field), PB X-PCI can achieve higher resolution and faster scans without the use of gratings. These *ex vivo* studies, together with the combined ultra-fast and high resolution capabilities that this technique has shown in other fields (48–50), prove PB X-PCI to be a very powerful candidate for the time-resolved 3D investigation of heart architecture.

This manuscript presents a proof-of-concept of a novel methodology for the study of heart architecture dynamics in rat models by combining PB X-PCI and a customized Langendorff system. The presented developments allowed to dynamically image beating hearts in 4D at an unprecedented 2.75 μm pixel size (10.6 μm spatial resolution) and 1 ms temporal resolution. Therefore, the presented methodology is able to characterize the structural mechanisms responsible for the cardiac cycle, including the consequences of remodeling diseases and the effect of clinically-relevant drugs.

## 2. Materials and methods

### 2.1. X-ray tomographic microscopy-compatible Langendorff system

**Figure 1:**
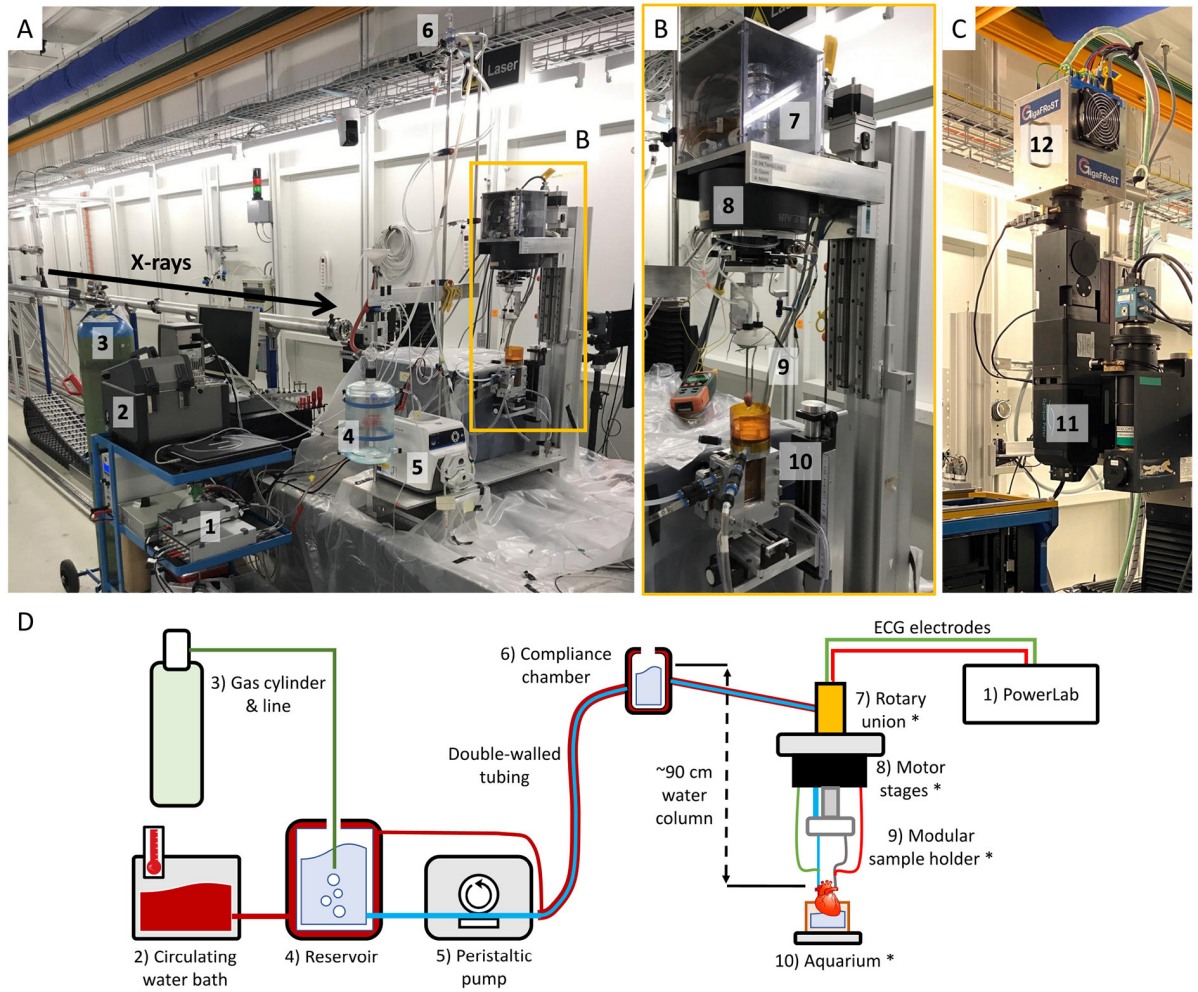
**(A)** X-ray tomographic microscopy-compatible Langendorff system at the TOMCAT beamline. (1) PowerLab data acquisition hardware. (2) Circulating water bath to keep the circuit at ~37°C. (3) 95% O_2_/5% CO_2_ gas bottle for perfusate oxygenation. (4) Perfusate reservoir. (5) Peristaltic pump circulating the perfusate up to the compliance chamber. (6) Compliance chamber. The height difference with the aorta determines the perfusion pressure (60 mmHg/~90 cm water column). **(B)** Close-up of the custom stage tower. (7) Rotary union system to ensure that the heart can be rotated without entangling tubes and wires. (8) Motor stage. (9) Modular sample holder, where the heart is attached at the ends of metallic aortic and left atrial cannulae. (10) Aquarium to keep the heart warm and collect the perfusate. **(C)** TOMCAT ultra-fast end-station. (11) High-numerical aperture 4x macroscope (53). (12) GigaFRoST detector (54). **(D)** Simplified sketch of the system components shown in **(A, B)**.

To achieve *in vitro* beating heart imaging, a Radnoti Rat Working Heart Apparatus was customized to fulfill the requirements of a synchrotron-based tomographic microscopy-compatible Langendorff setup. As illustrated in Figure 1 and listed in Table 1, the Langendorff system consists of a perfusate reservoir with an oxygenation line, a compliance chamber, connecting tubing and two pumps for the buffer solution and circulating water bath. The chambers and most tubing are double-walled, so that both the perfusate and the water bath can circulate. The difference in height between the compliance chamber and the aortic valve sets the pressure at which the perfusate will enter the coronary circulation (60 mmHg/~90 cm water column). To make the system compatible with tomography, a dedicated sample stage and aquarium were designed.

**Table 1:** Description and function of the hardware components of the tomographic microscopy-compatible Langendorff system, labeled with respect to Figure 1.

**Component and label**	**Description and function**
PowerLab (1)	Data acquisition instrument to record ECG, detector exposure and motor angular readback signals.
Circulating water bath (2)	Warms up water to the desired temperature and pumps it through the heating circuit of the system.
Double-walled tubing	Allows simultaneous buffer and warm water circuits, so that the buffer can arrive to the heart at the target temperature (~37^*o*^C).
Gas bottle and oxygenation line (3)	95% O_2_/5% CO_2_ gas mixture bottle and line to oxygenate the buffer solution and achieve appropriate pH values.
Reservoir (4)	Initial chamber, where the buffer solution is heated and oxygenated.
Peristaltic pump (5)	In charge of pumping the buffer from the reservoir to the compliance chamber.
Compliance chamber (6)	Highest chamber. The difference in height between the compliance chamber and the aorta sets the constant pressure at which the heart will be perfused (60 mmHg / ~90 cm water column).
Rotary union^*^ (7)	Both for liquid and electrical signal, ensures that tubes and cables do not entangle while tomographic rotation occurs. Includes heating patches and a power supply to ensure temperature control.
Motor stages^*^ (8)	Allow sample alignment (translation) and rotation.
Modular sample holder^*^ (9)	Easily-exchangeable 3D-printed cap with metallic cannulae made in-house, which will hold the heart in place. The custom metallic cannulae allow a compact design and conduct ECG signal without attaching the electrodes directly on the heart.
Aquarium^*^ (10)	Aluminium frame with Kapton walls that keeps the heart environment at the desired temperature and collects the perfusate dripping from the apex.

Customized components are marked by ^*^.

The sample stage consists of a Micos UPR 160-AIR (PI miCos, Eschbach, Germany) rotation motor with two perpendicular linear motors, which are placed top-bottom, so that hearts can hang from them. In addition, a Moflon MQR4-S12(N2325) (Moflon Technology, Sha Jing, China) multi-channel rotary union for liquid and electricity allows continuous delivery of the buffer solution to the heart without entangling the tubing during rotation. Electrical feed-through is also necessary to connect electrodes for electrocardiogram (ECG) measurements. The rotary union has its own heating system to avoid temperature loss. From the stage, modular in-house 3D-printed sample holders keep the metallic cannulae in position to bring the perfusate to the heart and hold it stable. Custom aortic and left atrial cannulae were metal by design in order to optimize their shape and be able to transmit electrical signal to the ECG electrodes, without damaging the heart.

The customized aquarium is an aluminum structure with kapton foil walls, which have negligible X-ray absorption. The aquarium is then filled with perfusate right until the level of the apex. This helps to preserve the tissue temperature and absorb the perfusate drops falling from the heart, thus reducing related heart motion.

Finally, a PowerLab 8/35 (AD Instruments Ltd) equipped with LabChart Pro 8 was used to measure the ECG signal of the heart, the exposure signal of the detector and the angular feedback of the rotation motor. Angular feedback was obtained by recording the sin/cos signals from the motor controller, as described by for multiscale imaging of human pancreatic tissue by Frohn et al. (51).

### 2.2. Langendorff preparation and perfusion protocol

Animal care and experimentation followed the European Convention for Animal Care and was approved by the Swiss Animal Welfare Authorities (Authorization number 75737/32784).

Wistar rats (3 males and 3 females, 12 weeks old) were obtained from Janvier Labs (France). The rats were housed and maintained at 22^*o*^C with a 12-h day/night cycle. Food and water were administered ad libitum.

The animals were anesthetized *via* intraperitoneal injection with a mixture of 125 mg/kg ketamine and 12.5 mg/kg xylazine, and provided with 100% medical oxygen *via* nose cone. Once loss of the pedal withdrawal reflex was verified, a rapid laparotomy and thoracotomy were performed. The heart was then removed from the chest cavity and placed in ice-cold physiologic buffer. The isolated, perfused preparation was then established by cannulating the aorta and left atrium. Even if the latter is not required for a Langendorff preparation, it was used for stabilization of the heart during tomographic measurements.

Perfusion was achieved with a modified Krebs-Henseleit solution (in [mM]: NaCl 118.0, KCl 4.7, KH_2_PO_4_ 1.2, CaCl_2_·2H_2_O 1.5, MgSO_4_·7H_2_O 1.2, Glucose 11.0, and NaHCO_3_ 25.0). The hearts started beating spontaneously. Their function was monitored until stabilization through ECG measurement, which was achieved by attaching one electrode to each cannula.

### 2.3. Image acquisition

The synchrotron-based X-ray tomography campaign was performed at the TOMCAT beamline of the Swiss Light Source (Paul Scherrer Institute, Villigen, Switzerland) (52). A PB X-PCI setup combined with the TOMCAT ultra-fast end-station was used to capture the dynamics of the cardiac architecture. As summarized in Table 2, a monochromatic X-ray beam at 21.9 keV energy and 220 cm propagation distance (sample-detector distance) were chosen. X-rays were converted to visible light by a LuAG:Ce 150 μm scintillator, magnified by a 4x high-numerical aperture macroscope (53) and recorded by the GigaFRoST detector (54). A total of 160,000 projections (1 ms exposure time, 2.75 μm voxel size, 5.54 x 2.75 mm^2^ field of view) were thus continuously acquired in continuous rotation over 720^*o*^. Assuming a constant cardiac cycle duration of 200 ms and considering the 1 ms exposure applied, 160,000 projections would then lead to a maximum of 800 projections per time-point within the heartbeat (a total of 800 heartbeats would occur during 160 s).

**Table 2:** Experimental dynamic acquisition parameters at the TOMCAT beamline.

**Parameter**	**Value**
Energy	21.9 keV
Propagation distance	220 cm
Pixel size	2.75 μm
Field of view	5.54 x 2.75 mm^2^
Field of view (pixels)	2,016 x 1,000
Projections, flats and darks	160,000; 300; 50
Exposure time	1 ms
Angular range	720^*o*^
Scintillator	LuAG:Ce 150 μm
Detector	GigaFRoST

While conventional tomography is commonly achieved with 180^*o*^ of rotation, a wider angular range was included to ensure a uniform angular distribution and compensate for potential cardiac dysfunction over time. In other words, if hearts start to lose contractility due to ischemia or radiation damage during a 180^*o*^ measurement, only a fraction of the projections (concentrated in a fraction of 180^*o*^) would be useful for reconstruction, thus leading to a very skewed angular distribution and the impossibility to achieve a successful tomogram.

### 2.4. Retrospective gating and image reconstruction

**Figure 2:**
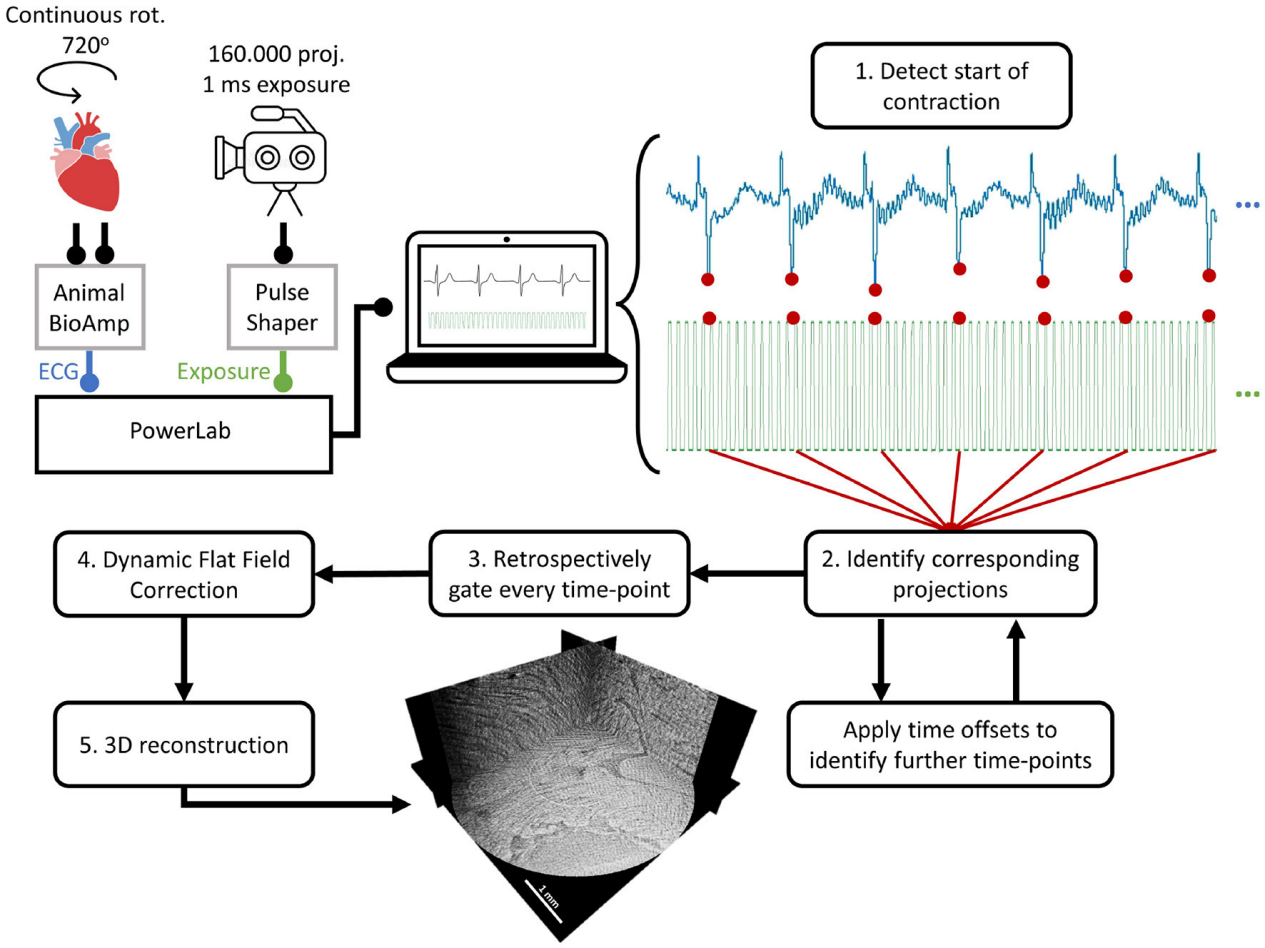
Sketch of the retrospective gating procedure. In dynamic scans, 160,000 projections (1 ms exposure) were acquired over 720^*o*^ of continuous rotation. The ECG of the beating heart was recorded by the PowerLab through an Animal BioAmp. Simultaneously, the exposure signal from the detector was sent through a pulse shaper to the PowerLab. For the retrospective gating, (1) the feature corresponding to the start of the contraction was detected in the ECG. (2) Then, all the corresponding projections acquired at that specific time-point were identified. To identify the projections in the rest of time-points, 1 ms offsets were applied until the time-series were completed. (3) With all projections assigned to each time-point, the data was gated and all individual datasets for every time-point were created. (4) All datasets were corrected using dynamic flat-field correction and (5) reconstructed to obtain the 3D volumes for every time-point in the heartbeat.

Retrospective gating (see sketch in Figure 2 was achieved by detecting the ECG feature corresponding to ventricular contraction and using it as a reference time-point to gate the time-series with a time resolution of 1 ms.

First, the detector exposure signal was used to select which exact ECG data points corresponded to each of the acquired projections. Then, by direct visual comparison of the ECG data points with their projections, the ECG feature indicating the start of ventricular contraction was found.

To detect this specific feature for every single heartbeat throughout the entire ECG, a single heartbeat signal was cross-correlated with the whole ECG. This allowed to detect the position of every heartbeat for independent processing. Then, within each heartbeat, the largest negative peak (corresponding to ventricular contraction) was detected.

Once this time-point was detected, it was used as the reference to calculate 190 additional time-points (1 ms each) by applying fixed time-offsets in each beat cycle. This was done under the assumption that ventricular contractions are reproducible in space and time. This is a demanding requirement at such high spatial and temporal resolutions and will affect the reconstruction quality when not holding true, as later discussed. In terms of heart physiology, this assumption should be correct unless contractility changes are induced by drugs or radiation damage.

Finally, all projection numbers with their corresponding time-bin number and acquisition angle were saved in a retrospective gating file, which was used as input to a script that generated every time-point dataset.

Due to the short exposure times and the use of a multilayer monochromator, the acquired projections were able to capture changes in beam uniformity, thus invalidating conventional flat-field correction based on the average of all flat-fields. To overcome this problem, a dynamic flat-field correction algorithm based on principal component analysis of the recorded flat-fields was applied to every dataset. In doing so, every individual projection was thus corrected with the most appropriate combination of eigen-flat-fields, as thoroughly detailed by Van Nieuwenhove et al. (55).

Phase information was retrieved applying the Paganin single-distance phase-retrieval algorithm (56). The δ/β ratio was finely tuned to a value of 200 from visual inspection of the reconstructions. Reconstructions were achieved by using the GridRec algorithm (57).

In order to investigate heartbeat reproducibility and the reduction on heart function over time, a window reconstruction method was applied. Reconstructions over 180^*o*^ were computed with 20^*o*^ steps from 0 to 360^*o*^, thus allowing observation of structural changes happening already during the first half of the scan. As a result, it was decided to keep dynamic reconstructions to the first 180^*o*^.

Spatial resolution was calculated using a Fourier analysis-based criterion (58) previously used in other phase contrast tomography studies (59, 60). Line profiles of all rows and columns for each image were obtained and their mean power spectral density was calculated. The power spectral density converges to the noise baseline, which can be converted to the corresponding spatial frequency, and thus to spatial resolution. These calculations lead to a spatial resolution of 3.86 pixels, or 10.6 μm. This is due to smoothing from optical components, low signal-to-noise ratio, image artifacts and phase reconstruction, among others (53, 58, 59).

Data visualization was achieved with the open-source software *ImageJ* (61), while retrospective gating and generation of time-series datasets was performed using in-house scripts in Python 3.4 and Matlab 2018a.

## 3. Results

In this proof-of-concept experiment, we used 6 rats with the goal to learn the possibilities offered by the setup and find potential technical challenges to be considered. Out of these, 1 female rat was successfully imaged, for which the corresponding results are presented in this section. The rest of rats allowed to improve the stability of the setup, optimize the acquisition scheme and understand the response of heart function to synchrotron radiation. A summary of technical recommendations can be found in Table 3.

**Table 3:** Summary of experimental considerations and corresponding recommendations.

**Topic**	**Description and recommendation**
Temperature control	Temperature losses can occur in unexpected parts of the Langendorff system. Make sure to warm up as many components of the setup as possible, including the hearts' surrounding air, and install temperature probes at different locations to detect the temperature leakage zones and correct as required.
Buffer pressure	As in the temperature case, points of pressure loss can be present. Make sure to detect them in advance with a pressure transducer and correct the water column height as required.
Buffer solution refill	The buffer solution's reservoir needs to be refilled periodically. Make sure to pre-warm and pre-oxygenate the new batch before adding it into the system to avoid temperature fluctuations that might affect heart function.
Surgery and perfusion	A rapid perfusion of the isolated heart is key to achieve a fully functioning sample. Perform the surgery as close as possible to the Langendorff system to minimize ischemia. In the reported experiment, an operation table was installed at the beamline (2 m from the setup).
Bubbles in circulation	For diverse reasons, air bubbles might enter in the Langendorff system and get stuck on the aortic valve, compromising coronary perfusion. During set up, make sure to remove all air from the tubing and perform motor rotations to extract as much air from the system as possible. Additionally, install extra tubing to allow possible bubbles to scape by creating a tubing T-junction right before the cannullae.
ECG quality	Place ECG electrodes so that optimal signal can be measured. Before proceeding, perform motor translations and rotations to ensure that these do not cause the electrodes to move nor signal distortions.
Heart motion	Due to the hanging position of the hearts, these will swing while beating and thus create unwanted motion. Try to minimize the swing by using 2 cannullae going as deep as possible into the heart, wihout damaging the cardiac valves or chambers. Since hearts are mechano-sensitive, adding physical constraints (i.e., a weighted net) will affect their function.
Cardiac function degradation	Due to radiation damage, heart function will degrade over time. Before deciding your acquisition scheme, make sure to understand the exposure that hearts can tolerate before heavily degrading. The evolution of the ECG signal is a good indicator.

**Figure 3:**
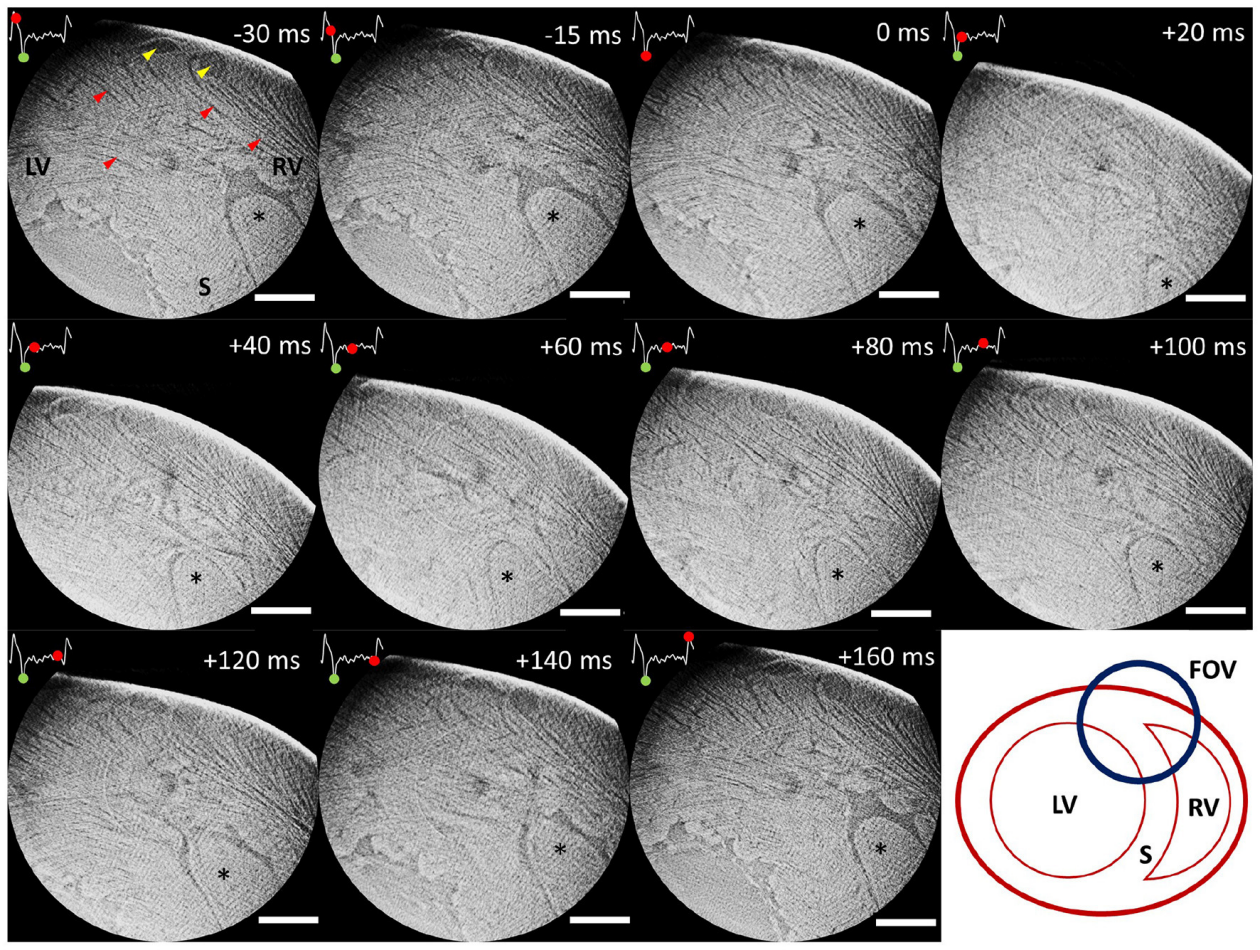
Time-series of an illustrative slice showing the region where left ventricular wall (LV), septum (S) and right ventricular wall (RV) join. Time is indicated using the start of the contraction as reference (0 ms). An ECG signal corresponding to a full heartbeat is included in every slice. The green dot on the ECG indicates the start of the contraction (reference time-point), while the advancing red dot corresponds to the exact reconstruction location. A right ventricular trabeculation has been marked (^*^) to clearly follow myocardial contraction and relaxation over time. Yellow arrows point at coronary vasculature, while red arrows point at cardiomyocyte aggregates. The bottom right image shows a sketch of the presented heart region. Scale bar is 1 mm.

**Figure 4:**
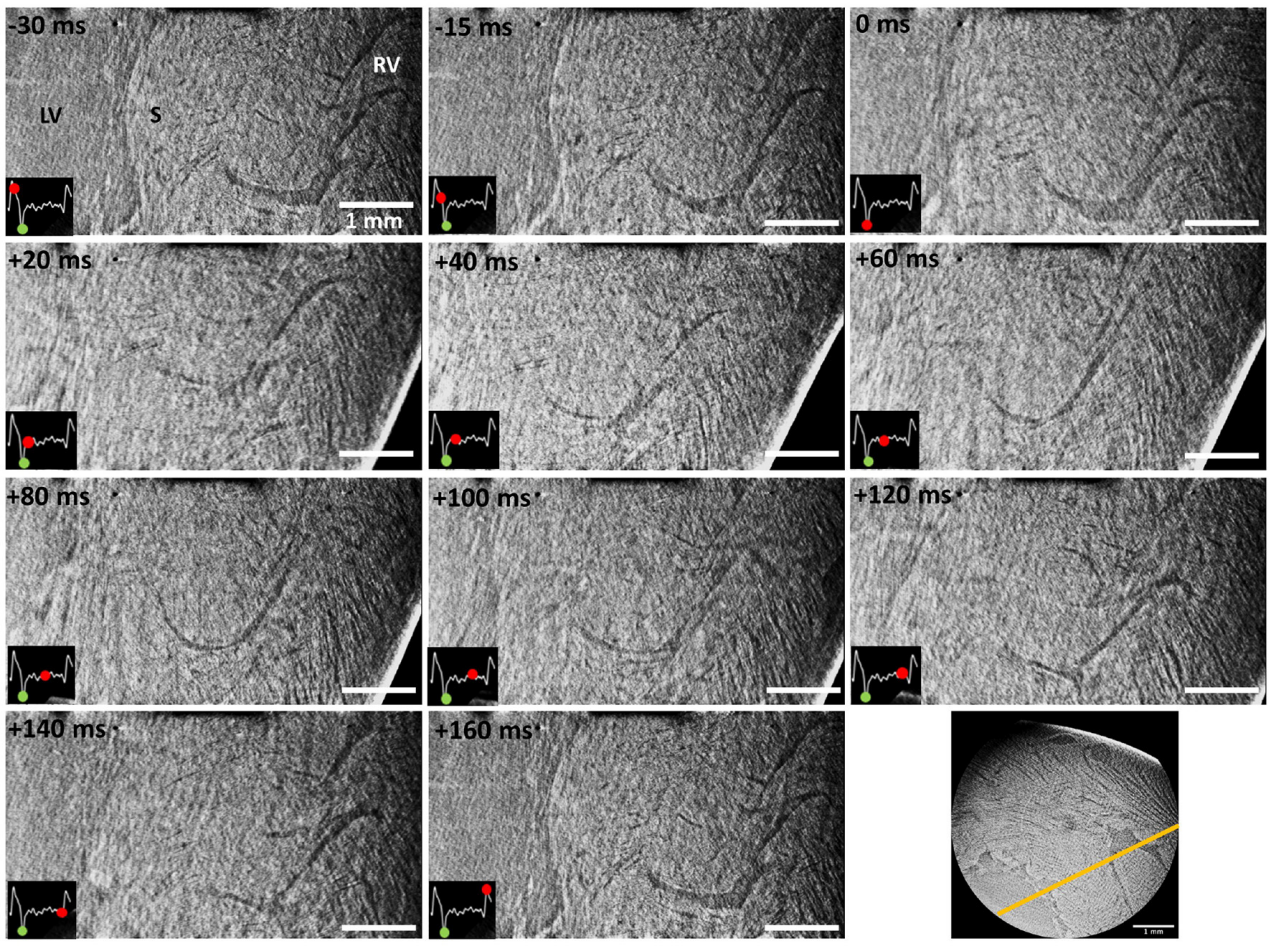
Time-series of an illustrative reslice (see bottom right, yellow line) from the scanned region including part of the left ventricular chamber (LV), septum (S) and right ventricular wall (RV). Time is indicated using the start of the contraction as reference (0 ms). An ECG signal corresponding to a full heartbeat is included in every slice. The green dot on the ECG indicates the start of the contraction (reference time-point), while the advancing red dot corresponds to the exact reconstruction location. Scale bar is 1 mm.

An illustrative time-series of the successfully imaged female heart is shown in Figure 3, Supplementary Video 1, using as reference time-point the start of the contraction (0 ms). This specific slice shows the region where the left ventricle, the septum and the right ventricle meet together. By following the marked structure (^*^, right ventricular trabeculation) through time, it can be noted how the right ventricular cavity is in a relaxed position during the negative time points (–30 and –15 ms). Then, systole starts at time 0 ms and ventricular contraction can be observed by the collapse of the cavity and the displacement of the heart, which was freely hanging in the air. As time evolves, the heart returns to its initial position and the cavity slowly opens again until the initial relaxation state. Figure 4, Supplementary Video 2 show two different resliced versions of the same time-series. In addition, Supplementary Video 3 presents sliding orthogonal views of a single time-point to demonstrate the 3D nature of the data.

The data presented in Figures 3, 4 was obtained using the projections acquired only during the first 180^*o*^, which were a total of 199 angular projections per time-point. This number comes from the fact that 199 heartbeats occurred during the first 180^*o*^. Such restriction was decided after observing data quality degradation over time as larger angular ranges were allowed. In this context, a window reconstruction method was applied to identify the sources of diminishing data quality.

**Figure 5:**
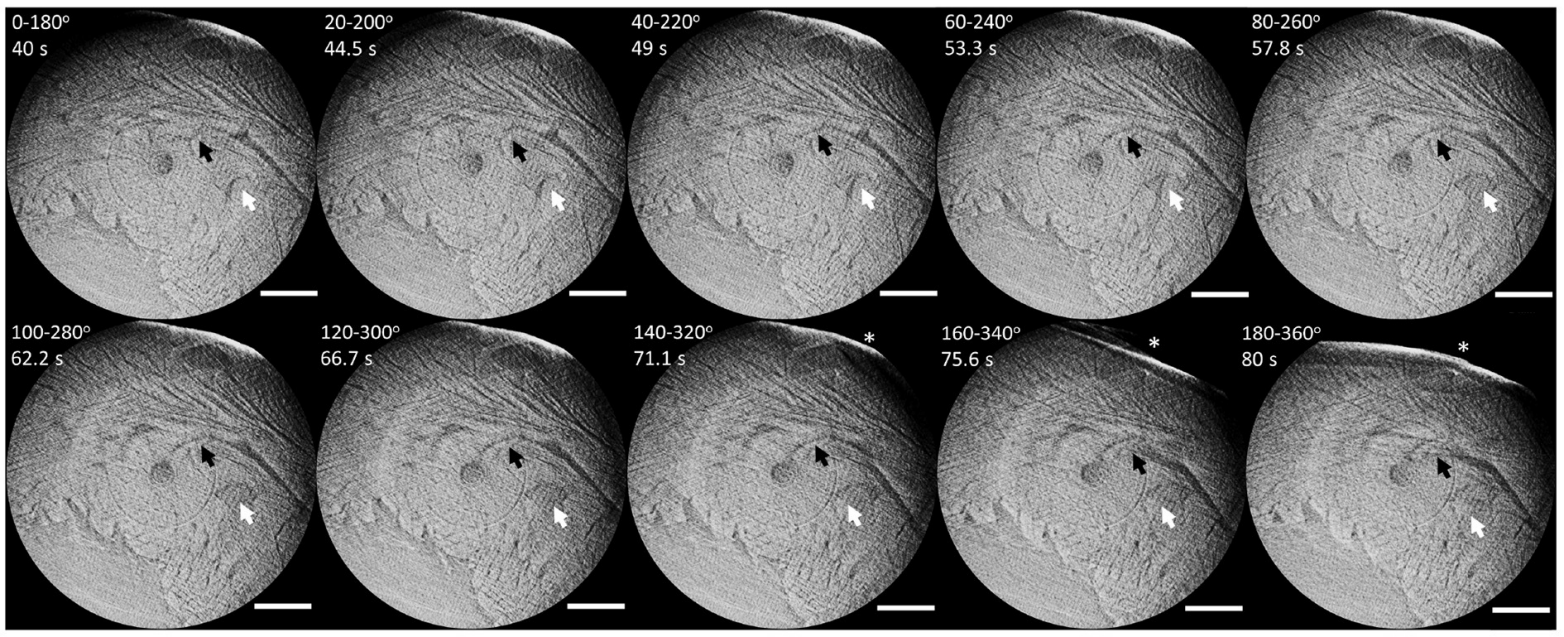
Series of representative window-reconstructed slices from 0–180^*o*^ to 180–360^*o*^ in 20^*o*^ steps. **Black** and **white** arrows indicate example structures progressively changing over time, thus indicating function and/or tissue degradation. The star marks a large variation on the heart surface, probably due to sliding perfusate. Scale bar is 1 mm.

Figure 5 shows the changes of a representative slice as the allowed reconstruction angles were varied. Each reconstruction corresponds to an angular range of 180^*o*^ but the starting angle is shifted by 20^*o*^ in every iteration. The arrows mark illustrative myocardial structures that change over time, thus indicating changes in cardiac function and/or structure over time. The largest changes can be observed during the last three reconstructions (marked by ^*^). These are most probably caused by the sliding and dripping of perfusate over the heart surface or a change in heart motion due to degradation. Since the observed changes are mainly progressive and slow, they are indicative of either ischemia (tissue shrinkage) and/or progressive dysfunction. These are probably caused by a combination of radiation damage, which leads to ischemia due to DNA damage and formation of radicals, local temperature increase, and from the *in vitro* preparation itself, which is not completely physiological.

In Figures 3, 5, Supplementary Videos 1, 3 central ring artifacts can be observed. These are typical from micro-CT measurements and can arise during flat and dark field correction, commonly from dirt particles in the scintillator or malfunctioning pixels, among other reasons. While ring removal could help eliminate these artifacts, they often cause (over)smoothing of the data, especially when trying to eliminate very sharp rings, which we wanted to avoid in the presented datasets.

## 4. Discussion

This manuscript presents the successful customization of an isolated, perfused heart system to allow *in vitro* synchrotron-based phase contrast tomographic experiments. The developed Langendorff setup has allowed dynamic imaging of a beating rat heart. This is a unique setup worldwide and, even if it has been specifically designed for the TOMCAT beamline, the same hardware customization principles and retrospective-gating technique could be applied at any other beamline or laboratory setup wishing to perform similar tomographic experiments.

Dynamic recordings of the beating heart were achieved at 2.75 μm pixel size (10.6 μm spatial resolution) and 1 ms temporal resolution, which is an unprecedented level of detail in this kind of heart measurements (3, 13–15). Reconstructions were obtained through retrospective gating using the ECG signal as reference. Illustrative time-series are presented in Figures 3, 4, Supplementary Videos 1, 2. While the motion observed is a combination of contraction and heart swinging from the cannulae, a clear wall thickening and contraction of the right ventricle can be observed in the data. The contraction process is observed to occur very rapidly within 20 ms after ventricular electrical activation, while full relaxation is slow and takes up to ~150 ms. All trials to reduce heart swinging were abandoned, since heart function was shown compromised due to the mechanosensitivity of the heart.

Due to the complexity of the experiment and the local field of view, dynamic data was currently limited to the qualitative observation of cardiomyocyte aggregates and larger cardiac structures, such as right ventricular trabeculation, cardiac wall and large vasculature. While the voxel size is 2.75 μm, the computed spatial resolution was 10.6 μm due to blurring and artifacts (e.g., low amount of projections). The challenge of the experiment lies in the efficient detection of X-rays while keeping the lowest exposure time possible, due to the rapid motion of the heart and cardiac function degradation over time. In this context, the developed setup included the available state-of-the-art equipment, such as the ultra-fast TOMCAT end-station (54) and high-numerical aperture 4x macroscope (53). This configuration allowed an exposure time as low as 1 ms. This means, therefore, that any movement larger than 1 pixel within 1 ms (2.75 μm/ms) will lead to motion artifacts in the reconstruction, as can be observed mostly during contraction (see Figures 3–5).

Similarly, function degradation leading to changes larger than a pixel will also introduce an artifact in the reconstruction. To tackle this issue, scans were recorded over several rotations with the goal to ensure enough angular sampling. After the experiment, 180^*o*^ window reconstructions with a step of 20^*o*^ were compared to assess how reproducible heartbeats were over time (see Figure 5). Seeing that after the first 180^*o*^ some changes observed in the reconstruction started to appear, it was decided to limit the retrospectively-gated reconstructions to the projections acquired in this initial 180^*o*^. This decision dramatically reduced the number of projections available for reconstruction to 199 per tomogram, which in turn reduced image quality and introduced streak artifacts. To compensate for these artifacts, the use of iterative reconstruction algorithms or new acquisitions schemes should be investigated.

Given these physical and (current) technological limitations, a deep quantitative analysis, such as cardiomyocyte orientation, would possibly lead to misleading results. Nevertheless, the dynamically reconstructed data shown in this manuscript are still very promising. In the future, the use of efficient lower magnification microscopes and detectors with faster read-out speed would allow to increase the area of the heart imaged at one time. Even if at the expense of a larger pixel size, this would improve image quality and temporal resolution, while trying to keep dose deposition as low as possible. Moreover, the addition of heart function sensors for e.g., coronary flow or LV cavitiy volume, would allow for a deeper understanding of heart behavior during measurements.

By approximating rat hearts as cylinders of skeletal muscle with 15 mm diameter and following the dose calculations described in other biomedical applications of synchrotron radiation (59, 62, 63), the dynamic acquisitions presented in this manuscript have been estimated to lead to total absorbed dose of ~18 kGy per scan (for the first 180^*o*^ tomography, ~4.5 kGy), which are typical values in synchrotron-based X-PCI (59, 64). Dose distribution is specially important for tomographic scans with reduced field of view in comparison with the total volume of the sample.

With the mentioned improvements, the dynamic data could be used to determine whether cardiomyocytes are organized in so called sheets and whether these slide on each other during the cardiac cycle (65). This is nowadays one of the main hypothesis that could describe the mechanics of the heartbeat and partially explain how the myocardium is able to thicken in a range of 28–50% during contraction, while individual cardiomyocytes can only thicken by ~8% (12, 66–69). Therefore, further developments of this setup have the potential to answer one of the main current questions in the field of cardiac anatomy and biomechanics.

Furthermore, the implementation of this setup opens the possibility to use cardiac remodeling models or even create them *in situ*, so that their very acute responses could be assessed. For instance, an ischemic model could be easily achieved by ligating the left anterior descendant artery while the heart is mounted, which is a commonly investigated animal model of cardiac infarct (37, 39, 70).

On top of that, the versatility of the setup allows to modify the perfusion solution given to the heart to change its behavior in a controlled manner. In that sense, cardioplegic arrest studies would be possible by using elevated *K*^+^ (diastolic arrest) or *Na*^+^-free *Li*^+^ Tyrode (systolic arrest) solutions (13, 14). In addition, iodine-based contrast agents could be added to the perfusate to investigate vascular behavior in real-time. Finally, specific drugs modifying heart contractility or causing vascular dilation-contraction could be easily investigated using this setup.

One of the main constraints of synchrotron experiments is the limited availability and duration of time slots. In the presented methodology, the bulk of the time is dedicated to the installation of the Langendorff system (~6 h), its synchronization with the beamline (~2 h) and stabilization of the perfusate temperature to the targeted ~37^*o*^C (~1 h). The time required for eventual refilling of perfusate is minimized, since it can be pre-warmed and -oxygenated through a parallel circuit while the system runs normally. Once these steps are achieved, the time needed between the application of anesthesia and a stable isolated, perfused heart is around 30 min. Acquisition of projections, flats and darks as applied in this study is well below 10 min. However, future more complex protocols as discussed above could extend the preparation and acquisition times. Therefore, one can assume that 1–2 h of experiment per heart are required, which would potentially allow to perform statistically supported studies.

This manuscript presents the customization of an isolated, perfused heart system compatible with tomographic PB X-PCI experiments at the TOMCAT beamline. The development of this type of setup opens the door to the dynamic study of heart architecture at a quasi-single cardiomyocyte level. This includes not only the study of normal heart structural dynamics, but also the possibility to create ischemic models *in situ* or to investigate the heart function effects of the administration of certain drugs in the perfusion solution. In the future, further analysis of this type of data could lead to more realistic computational models (71, 72) and a better understanding of the effects of remodeling in cardiac tissue deformation and heart function.

## Data availability statement

The raw data supporting the conclusions of this article will be made available by the authors, without undue reservation.

## Ethics statement

The animal study was reviewed and approved by Swiss Animal Welfare Authorities.

## Author contributions

HD, PG-C, SL, MS, BB, and AB designed the study. HD, CS, NM-C, MA, SL, and AB designed the experimental setup. HD, NM-C, MA, and SL handled the animals. HD and CS performed image and data processing. HD drafted the manuscript. All authors participated in the imaging experiments, revised, and contributed to the manuscript.
